# Analysis of the gut microbiota composition of myostatin mutant cattle prepared using CRISPR/Cas9

**DOI:** 10.1371/journal.pone.0264849

**Published:** 2022-03-04

**Authors:** Tong Wen, Chenyu Mao, Li Gao

**Affiliations:** Faculty of Biological Science and Technology, Baotou Teachers’ College, Baotou, Inner Mongolia, China; Huazhong University of Science and Technology, CHINA

## Abstract

Myostatin (*MSTN*) negatively regulates muscle development and positively regulates metabolism through various pathways. Although *MSTN* function in cattle has been widely studied, the changes in the gut microbiota due to *MSTN* mutation, which contribute to host health by regulating its metabolism, remain unclear. Here, high-throughput sequencing of the 16S rRNA gene was conducted to analyze the gut microbiota of wild-type (WT) and *MSTN* mutant (MT) cattle. A total of 925 operational taxonomic units (OTUs) were obtained, which were classified into 11 phyla and 168 genera. Alpha diversity results showed no significant differences between MT and WT cattle. Beta diversity analyses suggested that the microbial composition of WT and MT cattle was different. Three dominant phyla and 21 dominant genera were identified. The most abundant bacterial genus had a significant relationship with the host metabolism. Moreover, various bacteria beneficial for health were found in the intestines of MT cattle. Analysis of the correlation between dominant gut bacteria and serum metabolic factors affected by *MSTN* mutation indicated that *MSTN* mutation affected the metabolism mainly by three metabolism-related bacteria, *Ruminococcaceae_UCG-013*, *Clostridium_sensu_stricto_1*, and *Ruminococcaceae_UCG-010*. This study provides further insight into *MSTN* mutation regulating the host metabolism by gut microbes and provides evidence for the safety of gene-edited animals.

## Introduction

Yellow cattle are a characteristic resource of China, having 52 breeds, of which Qinchuan, Luxi, Nanyang, Jinnan, and Yanbian cattle have been domesticated and bred for thousands of years. They contain rich genetic resources and have rough feeding tolerance, strong stress resistance ability, strong adaptability, and tender meat. However, due to their long-term use as service cattle, common defects in these cattle, such as slow growth rate, underdeveloped rear-drive, slow fattening, weight gain, and low carcass production, cannot meet the requirements of international beef cattle.

Myostatin (*MSTN*), a member of the transforming growth factor β (TGF-β) superfamily, is highly expressed in skeletal muscle tissue and negatively regulates muscle growth [1]. *MSTN* mutation results in increased muscle mass in many species, including humans [2], sheep [3], mice [4], dogs [5], and cattles [6], without causing severe adverse effects. *MSTN* not only regulates muscle growth but also plays a vital role in fat metabolism [7], glucose metabolism [8, 9], bone development [10], and other metabolic processes [8].

*MSTN* has become a vital target gene for gene modification techniques in domestic animals because of its effects on skeletal muscle growth to enhance meat quality, and having a low fat percentage and a high lean yield percentage. *MSTN* mutant Luxi cattle, prepared at the Inner Mongolia University using Clustered regularly interspaced short palindromic repeats (CRISPR)/Cas9, showed an enhanced muscle phenotype with less body fat and more lean meat yield [11]. CRISPR/Cas9 technology is easy and effective for gene modification; however, it may result in problems including high off-target efficiency. Therefore, the safety of gene-edited animals is worthy of attention.

The gut microbiome comprises all microbial cells and related genetic material present in the gastrointestinal tract of the host. Numerous types of gut flora are closely associated with many physiological effects of the host [12–18]. Meanwhile, the dynamic equilibrium of gut flora is symbolic of gut health and the foundation for the healthy growth of the host. Therefore, the influence of intestinal microbial changes on the metabolic changes caused by *MSTN* gene mutations needs further investigation. Thus, clarifying the changes in the composition of intestinal microbes of the *MSTN* gene-edited cattle obtained using CRISPR/Cas9 technology is of great significance for the safety evaluation of gene-edited animals and the regulatory mechanism of *MSTN* gene mutants. In this study, 16S rRNA gene sequencing was used to identify the gut flora composition from fecal samples of wild type (WT) and *MSTN* mutant (MT) Luxi cattle bred under the same conditions. Furthermore, we analyzed the relationship between bacterial community and serum metabolic factors influenced by the *MSTN* mutant. The findings of this study provide further insight into the metabolic regulation mechanism of *MSTN* in cattle by affecting gut microbes and theoretical evidence on the relationship between *MSTN* and gut microbes and sheds new light on the safety evaluation for gene-edited animals.

## Materials and methods

### Ethical statement

The current study was performed as recommended on animal care and ethics in China. The Animal Ethics and Welfare committee (AEWC) of Baotou Teachers College and Inner Mongolia University approved the implementation of the project.

### Fecal sample collection

Fecal samples of three MSTN+/- and three WT Luxi cattle were collected. The MT cattle used in this study were prepared at the Inner Mongolia University using CRISPR/Cas9 as previously described [11]. Briefly, *MSTN*-targeted gRNA was designed and inserted into the CRISPR/Cas9 vector and then transfected into bovine fetal fibroblast cells. The *MSTN* mutant cells were selected and used as donor cells for somatic cell nuclear transfer to prepare MT embryos; hence, MT cattle were produced using embryo transfer technology. The *MSTN* gene editing protocol in cattle is illustrated in S1 Fig.

All cattle used in this study were females, approximately 24-month-old and fed on a local livestock farm as ordinary beef cattle. All fecal samples were collected from the reproductive tract of the cattle during the embryo transfer process, ensuring fresh and pollution-free feces, and were stored on dry ice for further analysis.

### Determination of serum biochemical indicators

Blood samples from the three WT and three MT cattle were collected and centrifuged for 10 min at 1000 rpm. The sera of MT and WT cattle for determining biochemical indicators were prepared. Serum biochemical indicators, including alanine aminotransferase (ALT), aspartate aminotransferase (AST), α-amylase (AMY), lactate dehydrogenase (LDHL) and lactate (LACT), were determined using a biochemical automatic analyzer (Hitachi 7020, Japan).

### DNA extraction, 16S rRNA sequencing, and data analysis

The DNA was extracted from the fecal samples using E.Z.N.A. Stool DNA Kit (D4015; Omega Bio-tek, Inc., Norcross, GA, USA) according to the manufacturer’s instructions. The V3-V4 hypervariable region of the bacterial 16S rRNA gene was amplified and sequenced, and the obtained raw data were analyzed as previously reported [19]. Operational taxonomic unit (OTU) level α-diversity indices, such as abundance-based coverage estimators (ACE), Chao1, Shannon index, and Simpson index, were computed to evaluate the diversity and richness of the bacterial composition. Non-metric multidimensional scaling (NMDS) based on weighted UniFrac distance matrices was used to define beta diversity. Linear discriminant analysis (LDA) effect size (LEfSe) analysis was used to indicate the significant ranking of plentiful modules in the MT and WT samples [20]. A logarithmic LDA score threshold of 2.0 was used to distinguish functional biomarkers. Figures were plotted using the R software [21] with the ‘ggplot2’ package. The functions of the bacteria with significant abundance (>1% in each group) were predicted using the Kyoto Encyclopedia of Genes and Genomes (KEGG) database. Redundancy discriminant analysis (RDA) biplots were performed to evaluate relative importance through the exploration of ‘explanatory’ and ‘response’ variables. Spearman’s correlations were used to prioritize marker species linking microbiota and serum biochemical indicators.

## Results

### Serum biochemical indicators of MT and WT cattle

Serum biochemical indicators involving ALT, AST, AMY, LDHL, and LACT were detected, results showed that significant differences were observed in all the detected serum biochemical indicators between WT and MT cattle (Table 1).

**Table 1:** Serum biochemical indicators of *MSTN* mutant (MT) and wild-type (WT) cattle.

Serum biochemical indicators	AST(U/L)	ALT(U/L)	AMY(U/L)	LDHL(U/L)	LACT(mmol/L)
WT1	53.3	18.8	18.4	1070	1.81
WT2	55.5	24.1	14.2	1009	1.24
WT3	51.4	22.0	11.2	1062	0.92
WT average	53.4±2.05	21.63±2.67	14.6±3.62	1047±33.15	1.32±0.45
MT1	65.7	26.9	23.1	1280	5.84
MT2	97	32.9	28.8	1378	9.82
MT3	75.4	30	23.1	1162	4.43
MT average	79.37±16.02	29.93±3.00	25±3.29	1273.33±108.15	6.69±2.79
*p* value	0.05	0.023*	0.021*	0.026*	0.030*

Data indicate mean ± standard deviations. Student’s t test was used to analysis the significance of differences between the two groups. n = 3.

*p < 0.05.

### Sequence statistics

**Fig 1:**
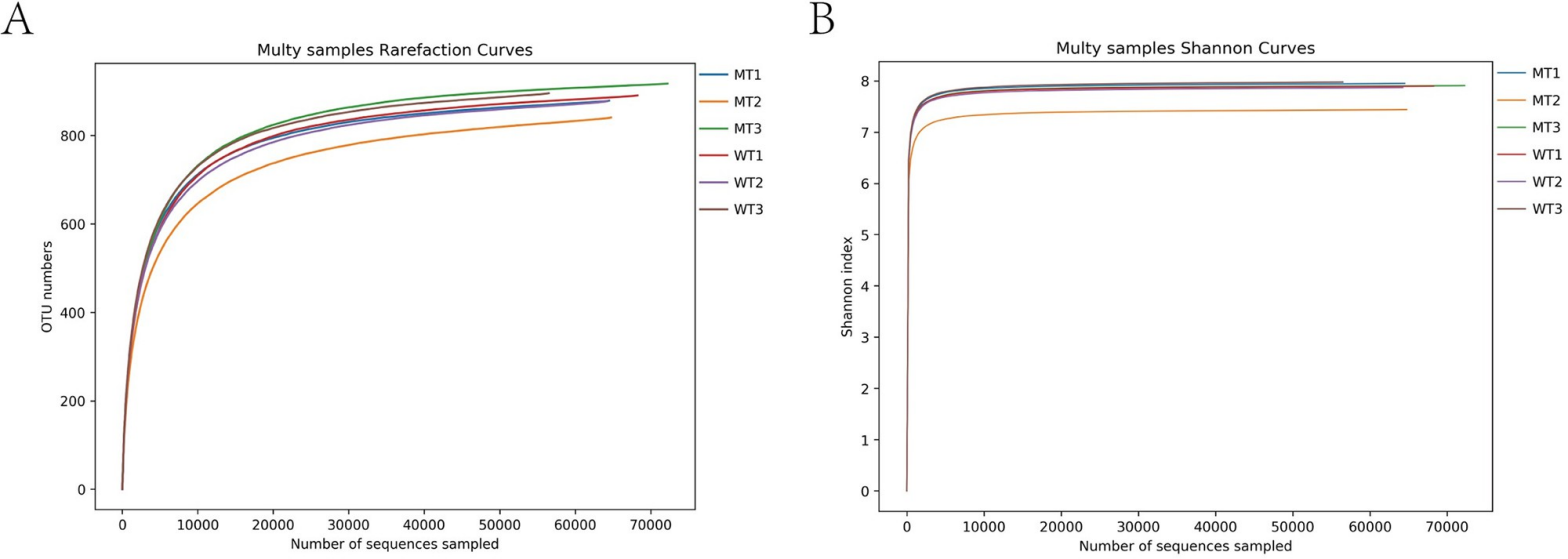
The Rarefaction curve (A) and Shannon diversity curve (B) of bacterial community in the analyzed samples. WT, wild type cattle; MT, *MSTN* mutant cattle.

A total of 501,216 effective sequences were acquired from the six cattle (three WT and three MT cattle), with 76,939–111,650 (mean 83,536 ± 13,784) effective sequences and 961 OTUs from each sample. These OTUs were classified into 11 phyla, 18 classes, 34 orders, 73 families, and 168 genera. Both the rarefaction curve and the Shannon index curve were almost flat, indicating that the bacterial diversity had achieved a platform. Further sequencing had no significant effect (Fig 1).

**Fig 2:**
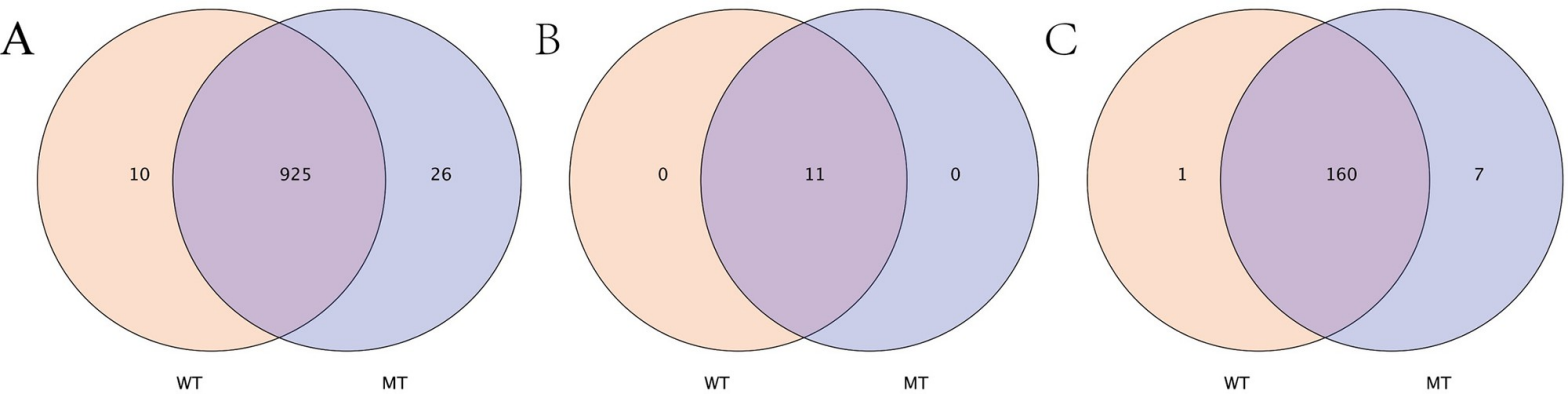
Venn diagram of the bacterial community in the analyzed groups. It shows the numbers of shared or not shared OTUs (A), phyla (B) and genera (C) by wild type cattle (WT) and *MSTN* mutant cattle (MT) individuals depending of overlaps.

A total of 925 OTUs were shared by cattle individuals of both WT and MT groups. Ten and 26 OTUs were present only in the gut microbiota of WT and MT cattle (Fig 2A). The 11 phyla detected were present in all cattle tested (Fig 2B). Further, the gut microbiota composition of the different cattle was analyzed. In addition to the 160 genera detected in both WT and MT cattle samples, one genus was unique in WT cattle, and seven genera were found only in MT cattle (Fig 2C).

### Alpha diversity and beta diversity analysis

**Fig 3:**
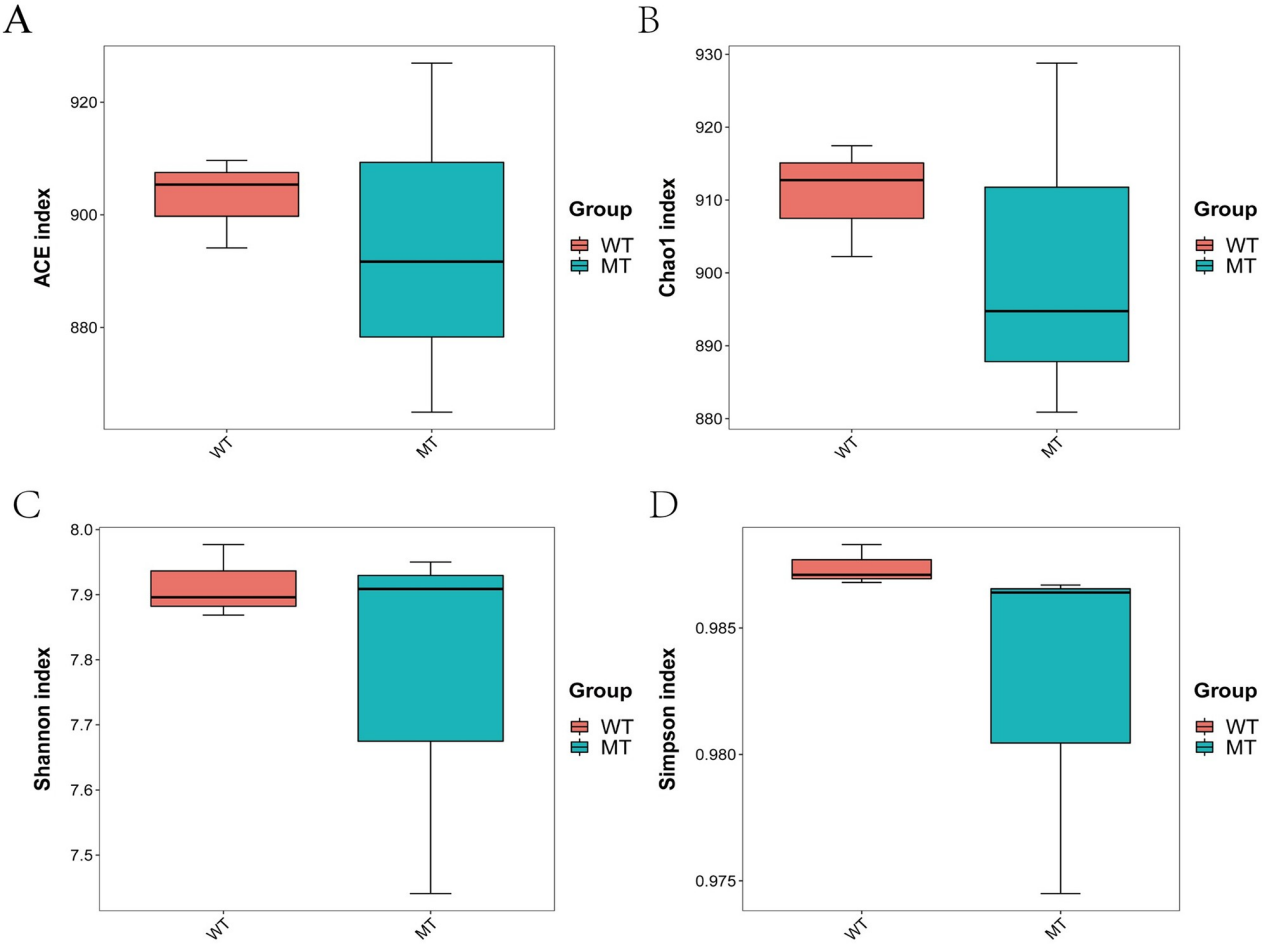
ACE index (A), Chao1 diversity (B), Shannon diversity (C) and Simpson indices (D) of bacterial populations in each sample. WT, wild type cattle; MT, *MSTN* mutant cattle.

In the current study, the alpha diversity analysis indicated that no significant differences existed in ACE (903.05 ± 4.63 and 894.53 ± 17.95), Chao1 (910.82 ± 4.49 and 901.48 ± 14.23), Shannon (7.91 ± 0.03 and 7.77 ± 0.16), and Simpson (0.9874 ± 0.0005 and 0.9825 ± 0.004) indices between WT and MT cattle (*p* > 0.05) (Fig 3).

**Fig 4:**
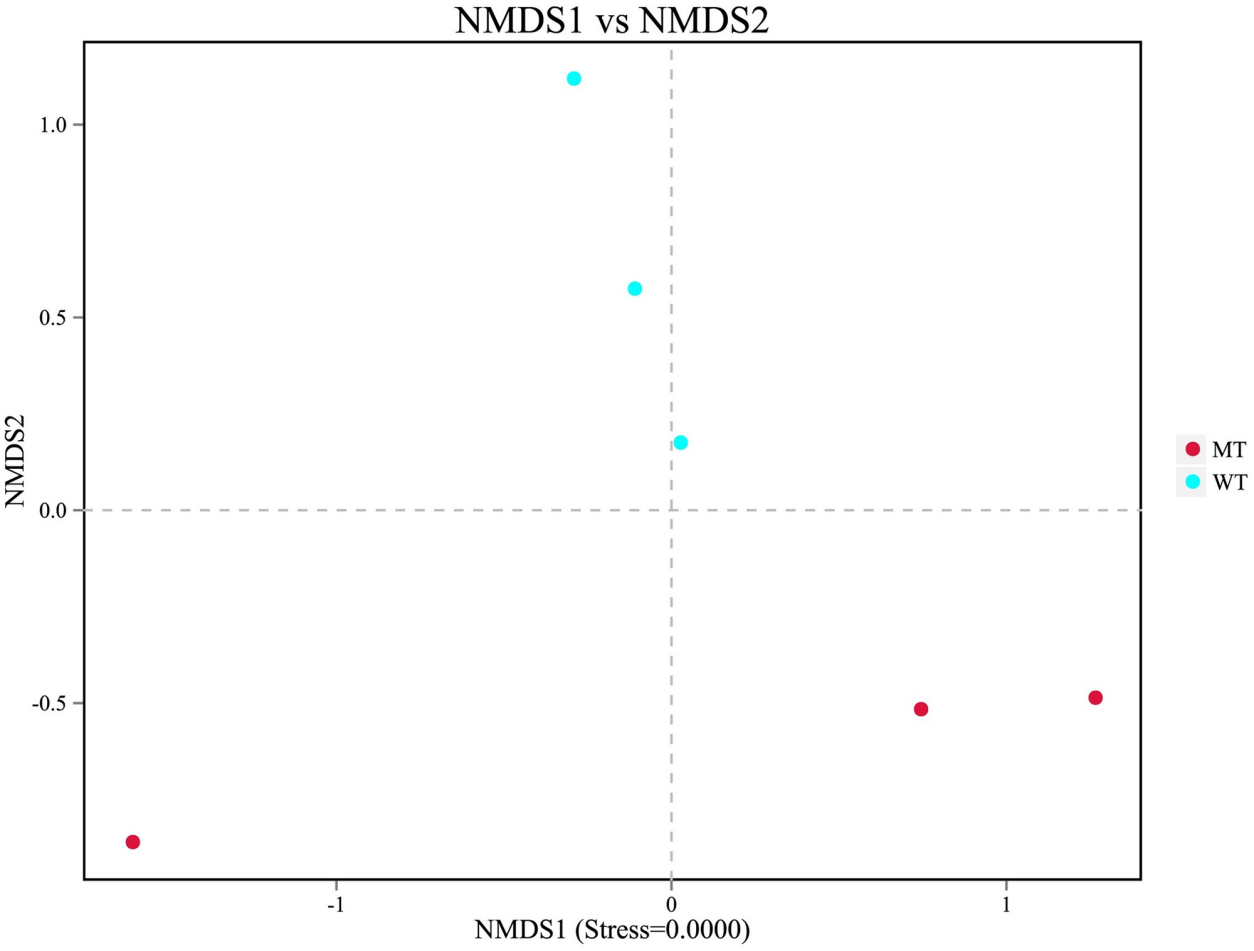
Results of the non-metric multidimensional scaling (NMDS) analysis. WT, wild type cattle; MT, *MSTN* mutant cattle.

The difference in the bacterial community between MT and WT cattle was evaluated using beta diversity analysis. The scatter plots were generated using NMDS based on weighted UniFrac distance. A significant clustering trend of fecal samples from the same type of cattle was revealed, indicating that MT led to the change in the microbial composition in the Luxi cattle (Fig 4).

### Global composition of gut bacterial communities of MT and WT cattle

**Fig 5:**
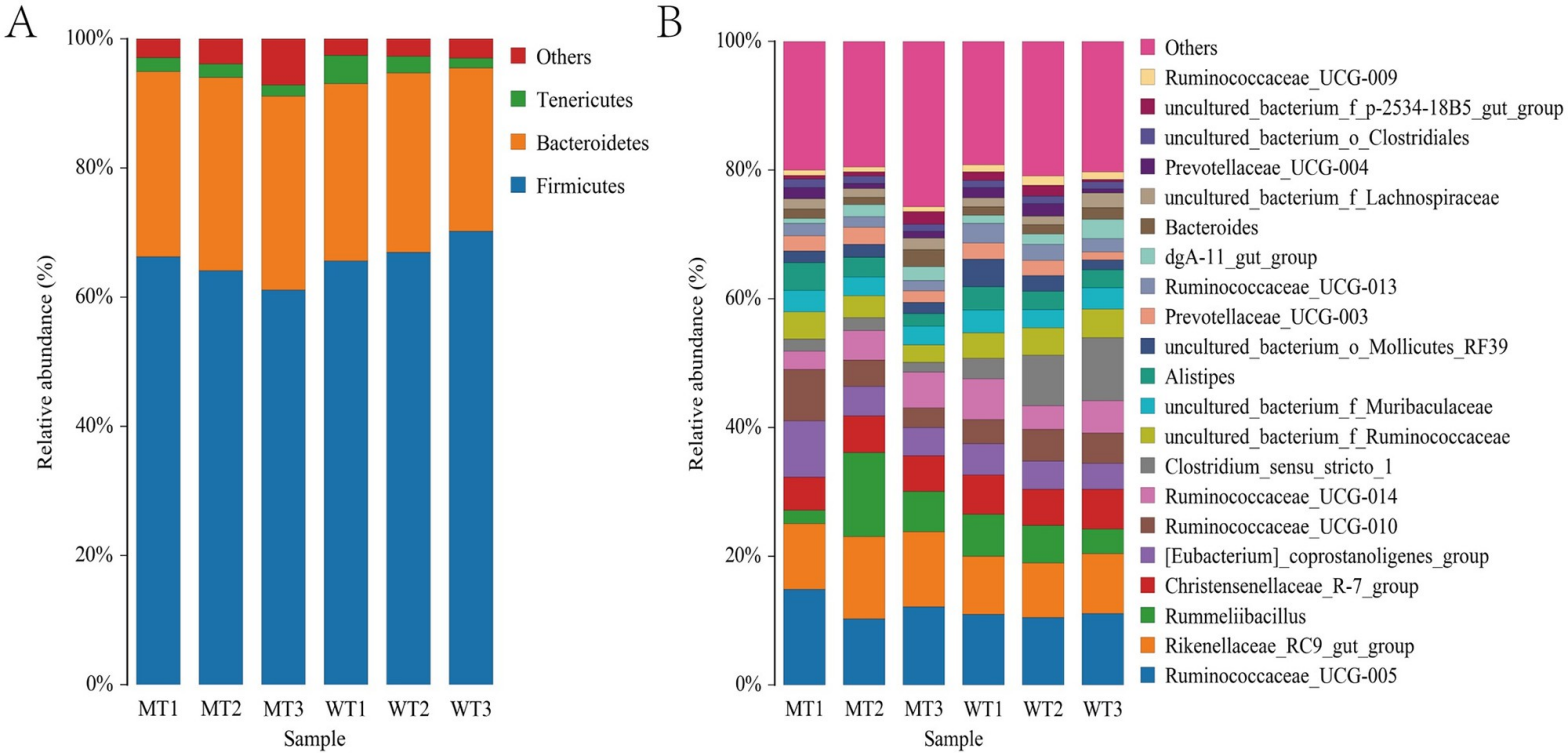
Bar graph of the relative bacterial abundance at phylum (A) and genus (B) level. The Bacterial those with relative abundance (%) over 1% were shown. Others, bacterial with a relative abundance of less than 1%. WT, wild type cattle; MT, *MSTN* mutant cattle.

After roughly analyzing the sequencing data, the bacterial composition in the fecal samples was further studied. Eleven phyla from the six fecal samples were identified, of which three showed an average relative abundance above 1% (Fig 5A), including Firmicutes, Bacteroidetes, and Tenericutes. The cumulative proportion of the three phyla was 96.29% for each sample. The gut microbiota of WT and MT cattle was dominated by Firmicutes.

The bacterial composition of the samples was analyzed at the genus level. A total of 168 bacterial genera from the six fecal samples were identified, of which 21 showed an average relative abundance above 1%. These were *Rikenellaceae_RC9_gut_group*, *Ruminococcaceae_UCG-005*, *Rummeliibacillus*, *Christensenellaceae_R-7_group*, *Ruminococcaceae_UCG-010*, *[Eubacterium]_coprostanoligenes_group*, *Clostridium_sensu_stricto_1*, *uncultured_bacterium_f_Ruminococcaceae*, *Ruminococcaceae_UCG-014*, *uncultured_bacterium_f_Muribaculaceae*, *Alistipes*, *Prevotellaceae_UCG-003*, *uncultured_bacterium_o_Mollicutes_RF39*, *Ruminococcaceae_UCG-013*, *dgA-11_gut_group*, *Bacteroides*, *uncultured_bacterium_f_Lachnospiraceae*, *Prevotellaceae_UCG-004*, *uncultured_bacterium_o_Clostridiales*, *uncultured_bacterium_f_p-2534-18B5_gut_group*, and *uminococcaceae_UCG-009* (Fig 5B). The dominant genus in both WT and MT cattle was *Ruminococcaceae_UCG-005*. Furthermore, its closely related species, *Ruminococcaceae_UCG-009*, was dominant (>1%) in WT cattle (1.23 ± 0.16%); however, its relative abundance in MT cattle was <1% (0.80 ± 0.05%).

### Differences between gut microbial communities of MT and WT cattle

**Fig 6:**
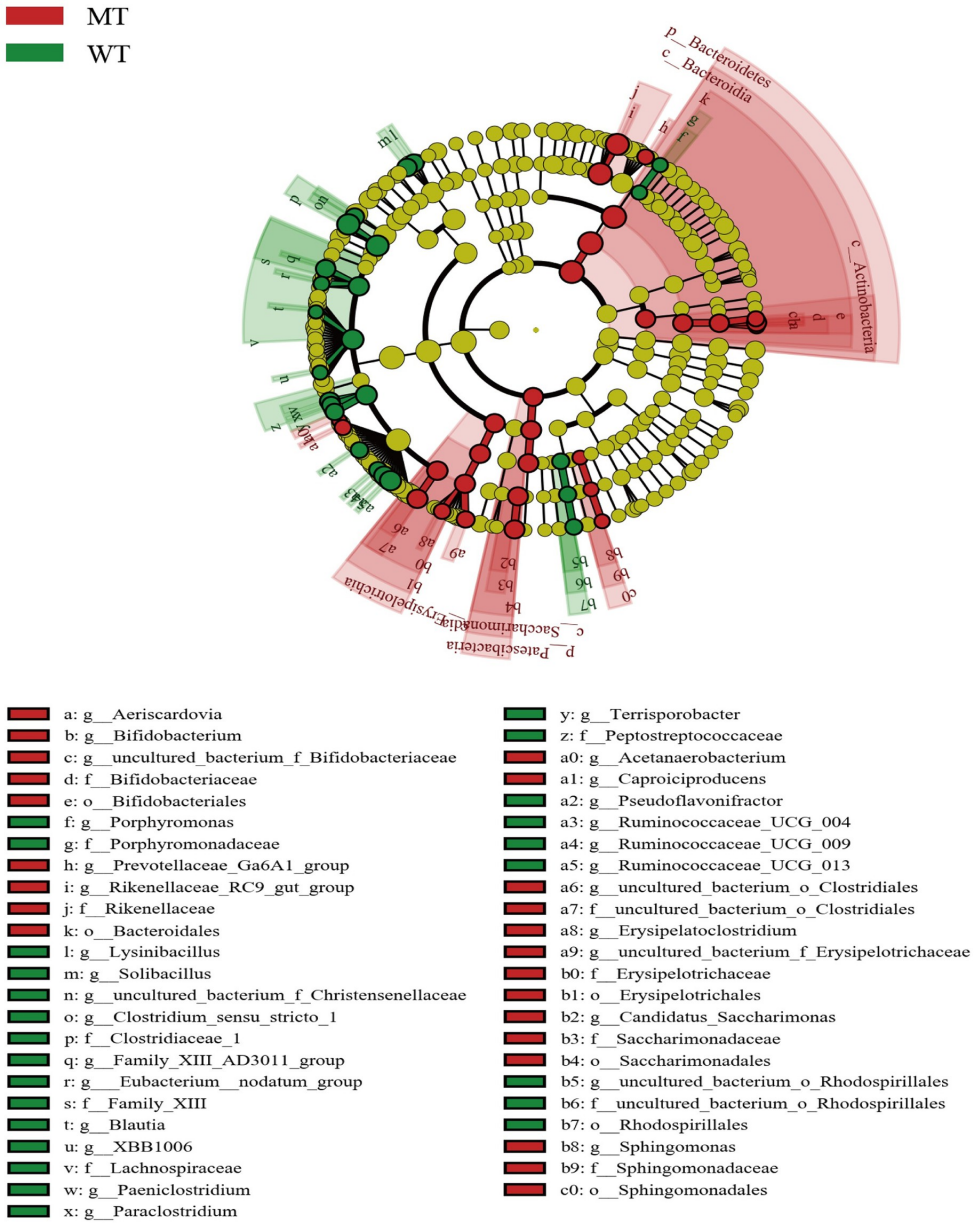
Linear discriminant analysis effect size (LEfSe) analysis. The microbial species which had significant differences in WT and MT cattle. Red presents the MT group, green presents the WT group. The classification at the level of genus, family, order, class and phylum were exhibited from the outside to the inside (LDA score > 2.0).

The gut microbiota composition of WT and MT cattle was analyzed to identify whether *MSTN* mutation affects the gut bacterial communities of the cattle. Substantial differences were observed in gut flora between WT and MT cattle. At the genus level, the presence of *Caproiciproducens*, *Erysipelatoclostridium*, *Prevotellaceae_Ga6A1_group*, *uncultured_bacterium_f_Bifidobacteriaceae*, *uncultured_bacterium_o_Clostridiales*, *uncultured_bacterium_f_Erysipelotrichaceae*, *Acetanaerobacterium*, *Aeriscardovia*, *Candidatus_Saccharimonas*, *Bifidobacterium*, *Sphingomonas*, and *Rikenellaceae_RC9_gut_group* were substantially higher in MT cattle than in WT cattle. However the presence of *Ruminococcaceae_UCG-013*, *Clostridium_sensu_stricto_1*, *Solibacillus*, *Lysinibacillus*, *Ruminococcaceae_UCG-009*, *Family_XIII_AD3011_group*, *Paraclostridium*, *Blautia*, *Porphyromonas*, *uncultured_bacterium_f_Christensenellaceae*, *Terrisporobacter*, *Pseudoflavonifractor*, *XBB1006*, *Paeniclostridium*, *Ruminococcaceae_UCG-004*, *[Eubacterium]_nodatum_group*, and *uncultured_bacterium_o_Rhodospirillales* were substantially lower in MT cattle than in WT cattle (Fig 6) (LDA > 2.0, *p* < 0.05).

### Relationship between serum biochemical indicators and gut microbial communities

**Fig 7:**
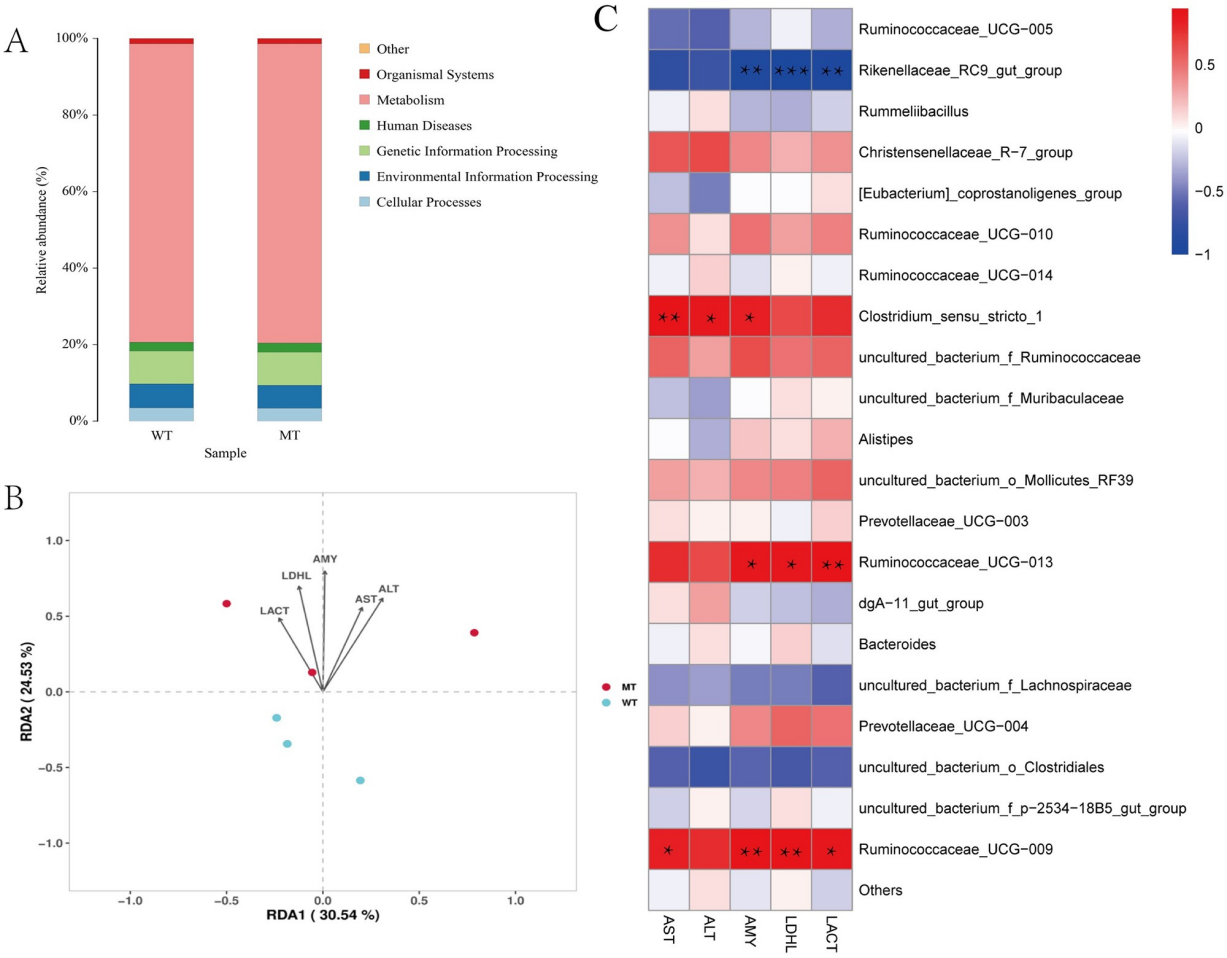
Relationship between serum biochemical indicators and gut microbial communities. (A) Bacterial community functional prediction by KEGG database. (B) Redundancy discriminant analysis (RDA) biplot indicating relationships between bacterial communities and serum biochemical indicator variables. (C) Relationship between serum biochemical indicator and gut microbial communities. Red/blue: positively/negatively correlation.*p < 0.05; **p < 0.01; ***p < 0.001.

The functions of the bacteria with significant abundance (>1% in each group) of the MT and WT cattle were predicted using the KEGG database. The primary functions of these bacteria were related to metabolism (Fig 7A). Several serum biochemical indicators, including ALT, AST, AMY, LDHL, and LACT were upregulated in MT cattle (Table 1). Further, the relationship between the serum biochemical indicators and the gut microbial community of WT and MT cattle was analyzed using the redundancy discriminant analysis (RDA). The RDA analyses indicated that the influence of the serum biochemical indicators differed on bacterial communities (Fig 7B). The first axis illustrated 30.54% and the first and second axes together illustrated 55.07% of the diversity in sample–serum biochemical indicators connections. The most related serum biochemical indicator to bacterial composition was AMY, and ALT and LDHL were significantly related. All serum biochemical indicators were closely related to the bacterial communities in MT cattle (Fig 7B).

The relevance between microbial species abundances and serum biochemical indicators was examined to identify marker species linking microbiota and serum biochemical indicators. Spearman’s rank correlation coefficients between abundant microbial species (>1% in each group) and serum biochemical indicators of the MT and WT cattle were determined (Fig 7C). *Clostridium_sensu_stricto_1* and *Ruminococcaceae_UCG-009* (*p* < 0.05) were significantly positively correlated to AST. *Clostridium_sensu_stricto_1* (*p* < 0.05) showed a significant positive relationship with ALT. *Clostridium_sensu_stricto_1*, *Rikenellaceae_RC9_gut_group*, *Ruminococcaceae_UCG-013*, and *Ruminococcaceae_UCG-009* (*p* < 0.05) were significantly correlated to AMY, of which *Rikenellaceae_RC9_gut_group* was negatively correlated, whereas the others were positively correlated. *Rikenellaceae_RC9_gut_group*, *Ruminococcaceae_UCG-009*, and *Ruminococcaceae_UCG-013* (*p* < 0.05) were significantly related to LDHL, of which *Rikenellaceae_RC9_gut_group* was negatively correlated, whereas the others were positively correlated. *Rikenellaceae_RC9_gut_group*, *Ruminococcaceae_UCG-013*, and *Ruminococcaceae_UCG-009* (*p* < 0.05) were significantly related to LACT, of which *Rikenellaceae_RC9_gut_group* were negatively correlated, and the others were positively correlated.

## Discussion

*MSTN*, a TGF-β superfamily member, functions as a negative regulator during muscle growth in many species [1–6]. Therefore, extensive efforts have been made to efficiently inhibit *MSTN* expression for achieving muscle-improved animals [22–25]. To our knowledge, all *MSTN* knockout animals have developed skeletal muscle development. Continued in-depth study of regulation mechanisms has shown that *MSTN* regulates the metabolism of the host, including muscle development, fat metabolism [7], glucose metabolism [8, 9], bone development [10], and other metabolic processes.

The bacterial microbiota in the host plays vital roles in the energy and nutrition metabolism, reproduction, and immune homeostasis of the host [26]. Concerning metabolism, they provide enzymes that are not encoded by the host genome, such as the enzymes to digest complex compounds, including polyphenols and polysaccharides, and synthesize vitamins. In contrast, the gut microbial composition can reflect the health of the body. To date, the composition of the intestinal flora of most MT animals remains unknown. Only one study has analyzed the intestinal flora in MT pigs [27]. In this study, high-throughput sequencing of the 16S rRNA gene was performed to analyze the composition of the gut microbial community of MT cattle. Furthermore, the correlation between the changes in the gut microbial community and serum physiology of the host caused by *MSTN* mutation was discussed. This study provides a research basis for evaluating the health status of MT cattle intestinal microflora and the effect of *MSTN* mutation on the gut microbial composition.

The diversity and richness of the observed OTUs showed no significant difference between the fecal samples of MT and WT cattle, indicating that the *MSTN* mutation had no significant influence on the richness and diversity of gut microbiota, which is consistent with the results on the MT pigs [27]. NMDS based on weighted UniFrac distance showed significant differences in gut microbiota composition between the MT and WT cattle, indicating that *MSTN* mutation influenced the gut microbiota.

At the phylum level, 11 phyla in the six fecal samples were identified, and Firmicutes, Bacteroidetes, and Tenericutes showed an average relative abundance above 1%. Firmicutes and Bacteroidetes were core microbiota of both MT and WT cattle, which is consistent with the results on MT pigs [27]. Previous studies in humans reported that an elevated Firmicutes/Bacteroidetes (F/B) ratio was associated with obesity [28, 29]. In this study, the F/B ratio was lower in MT cattle (2.16 ± 0.14) than that in the WT cattle (2.53 ± 0.21). Although the difference is not significant, it can partly explain why *MSTN* mutation promoted fat metabolism and inhibited its formation as reported before [30]. In other words, *MSTN* mutations can regulate metabolism by regulating changes in the gut flora community.

At the genus level, 168 genera in the six fecal samples were identified, of which 21 showed an average relative abundance above 1%. Most of them were related to metabolism by function prediction, of which some were butyrate-producing gut bacteria, such as *Ruminococcaceae_UCG-005* [31], *Ruminococcaceae_UCG-014*, *Ruminococcaceae_UCG-010*, *Ruminococcaceae_UCG-013*, *uncultured_bacterium_o_Mollicutes_RF39* [32], and *uncultured_bacterium_o_Clostridiales* [33]; others were cellulose-degrading bacteria, such as *Ruminococcaceae_UCG-005*, *Rummeliibacillus* [34], *uncultured_bacterium_f_Ruminococcaceae*, and *Prevotellaceae_UCG-004* [35]. These bacteria are strongly associated with obesity, food metabolism, and serum metabolites. Furthermore, a small number of high-abundance bacteria, such as *Bacteroides*, associated with human diseases, such as colorectal inflammation [36], were observed. In addition, *Bacteroides* are a commonly occurring flora in the living environment of animals [37], indicating that the living environment plays a vital role in the gut microbial composition.

The abundance of some bacteria was significantly higher in MT cattle than those in WT cattle. Most of the abundant bacteria such as *Caproiciproducens* [38], *Acetanaerobacterium* [39], and *Sphingomonas* [40] are related to strengthening metabolism function, such as carbohydrate utilization and butyrate production, to protect the intestine structure and increase the antioxidant enzyme concentration. Some microorganisms were those beneficial to intestinal health, such as *uncultured_bacterium_f_Bifidobacteriaceae* [41], *Aeriscardovia* [42], and *Bifidobacterium* [41], which play a vital role in maintaining the balance of gut flora. A high proportion of beneficial bacteria represents healthy hosts [18], and our results indicate that the MT cattle were healthier than WT cattle, providing evidence for the safety of gene-edited animals. *MSTN* mutation leads to a decrease in fat content and an increase in the lean meat rate, which enhances the metabolism efficiency and reduces type 2 diabetes risk [43]. In our study, we found that the abundance of *Pseudoflavonifractor*, a type 2 diabetes-related flora, was decreased in MT cattle, indicating that *MSTN* can regulate metabolism by regulating intestinal flora. Some flora associated with intestinal inflammation was increased in MT cattle, such as *Erysipelatoclostridium* [44], *Prevotellaceae_Ga6A1_group* [45], and *Candidatus_Saccharimonas* [46], whereas some flora associated with intestinal inflammation was decreased in MT cattle, such as *Paraclostridium*, *Porphyromonas*, *Terrisporobacter*, and *Paeniclostridium*. Therefore, in this study, the effect of *MSTN* mutation on intestinal inflammation could not be determined. The results in MT pigs showed that *MSTN* mutation leads to a relative reduction in the inflammatory response [27]; however, it needs further investigation in MT cattle.

In this study, we found that *MSTN* mutation was correlated with serum metabolic factors. All serum biochemical factors were related to enhanced metabolism, indicating that *MSTN* mutation leads to increased metabolism, which was consistent with a previous study [47]. Previous studies have shown that *Rikenellaceae_RC9_gut_group* was positively correlated with HFD-induced “harmful indicators” and negatively correlated with “beneficial indicators” [48]. In our study, *Rikenellaceae_RC9_gut_group* was negatively correlated with AMY, LDHL, and LACT, suggesting that *MSTN* mutation was negatively correlated with HFD-induced “harmful indicators” and positively correlated with “beneficial indicators.” In addition, *Clostridium_sensu_stricto_1* was positively correlated with AST, ALT, and AMY, *Ruminococcaceae_UCG-013* was positively correlated with AMY, LDHL, and LACT, and *Ruminococcaceae_UCG-010* was positively correlated with AST, AMY, LDHL, and LACT. The three abovementioned bacteria were all indicators for improving metabolic intensity [39], which is consistent with the phenotype of *MSTN* mutation and indicates that *MSTN* mutation mainly affected the metabolism by regulating these three bacteria.

## Conclusion

In this study, high-throughput sequencing of the 16S rRNA gene was performed to analyze fecal samples of WT and MT cattle. *MSTN* mutation had no remarkable influence on the diversity and richness of the gut microbiota in Luxi cattle. However, *MSTN* mutation influenced the composition of gut microbiota. The most abundant bacterial genus had a significant relationship with host metabolism. Moreover, the abundance of microorganisms beneficial for the health in the intestine of MT cattle was higher than in the WT cattle. Analysis of the correlation between bacteria and serum metabolic factors affected by *MSTN* mutation indicated that *MSTN* mutation affected the metabolism mainly by three metabolism-related bacterial, *Ruminococcaceae_UCG-013*, *Clostridium_sensu_stricto_1*, and *Ruminococcaceae_UCG-010*. The findings of this study provide further insight into *MSTN* mutation regulating host metabolism via gut microbes and theoretical evidence for the connection between *MSTN* and gut microbes. In addition, this study demonstrates a novel way to evaluate the safety of gene-edited animals.

## Supporting information

S1 Fig The evidences of gene editing of MSTN of the MT cattle used in the study. (DOCX)

S1 Appendix Bacteria_MT1_raw_1.fq. (GZ)

S2 Appendix Bacteria_MT1_raw_2.fq. (GZ)

S3 Appendix Bacteria_MT2_raw_1.fq. (GZ)

S4 Appendix Bacteria_MT2_raw_2.fq. (GZ)

S5 Appendix Bacteria_MT3_raw_1.fq. (GZ)

S6 Appendix Bacteria_MT3_raw_2.fq. (GZ)

S7 Appendix Bacteria_WT1_raw_1.fq. (GZ)

S8 Appendix Bacteria_WT1_raw_2.fq. (GZ)

S9 Appendix Bacteria_WT2_raw_1.fq. (GZ)

S10 Appendix Bacteria_WT2_raw_2.fq. (GZ)

S11 Appendix Bacteria_WT3_raw_1.fq. (GZ)

S12 Appendix Bacteria_WT3_raw_2.fq. (GZ)

S1 Raw images (PDF)
