# Supplementary material for: Analysis of the gut microbiota composition of myostatin mutant cattle prepared using CRISPR/Cas9
Source: PLoS One. 2022 Mar 4;17(3):e0264849. doi: 10.1371/journal.pone.0264849 (PMC8896723; doi:10.1371/journal.pone.0264849)
Supplement: S1 Fig — (DOCX) [file pone.0264849.s001.docx]

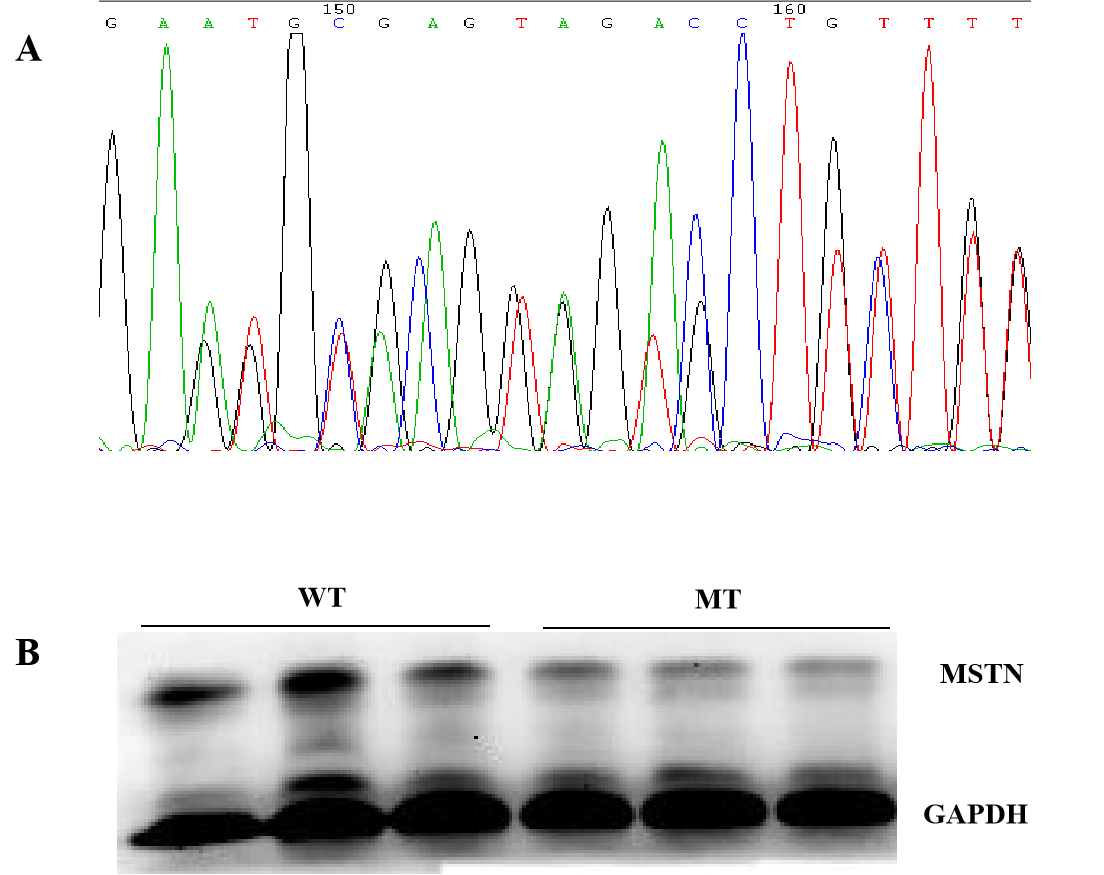


**Figure S1**. The evidences of gene editing of MSTN of the MT cattle used in the study. A. The identification results at the gene level of the MT cattle. B.The protein expression level of MSTN in muscle tissue of WT and MT cattle.
