# Supplementary material for: Analysis of the gut microbiota composition of myostatin mutant cattle prepared using CRISPR/Cas9
Source: PLoS One. 2022 Mar 4;17(3):e0264849. doi: 10.1371/journal.pone.0264849 (PMC8896723; doi:10.1371/journal.pone.0264849)
Supplement: S1 Raw images — (PDF) [file pone.0264849.s014.pdf]

S1_raw_images

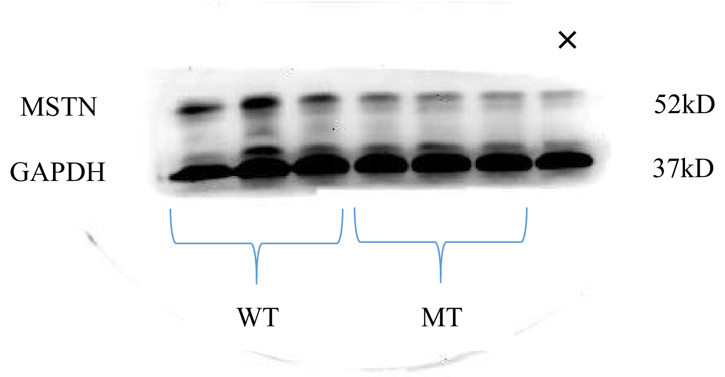

Figure S1B was generated from this raw figure.
Raw figure was captured by a Tanon (Shanghai, China) gel imaging system.
Anti-body used for Western blot was: anti-MSTN (sc-134345; Santa Cruz, USA), anti-GAPDH(sc-47724; Santa Cruz, USA).
